# Long-Term Outcomes of Radial Keratotomy, Laser In Situ Keratomileusis, and Astigmatic Keratotomy Performed Consecutively over a Period of 21 Years

**DOI:** 10.1155/2015/592495

**Published:** 2015-03-19

**Authors:** Priyanka Chhadva, Florence Cabot, Anat Galor, Sonia H. Yoo

**Affiliations:** ^1^University of Miami Miller School of Medicine, Bascom Palmer Eye Institute, Miami, FL 33136, USA; ^2^Ophthalmic Biophysics Center, McKnight Vision Center, Miami, FL 33136, USA; ^3^Miami Veterans Administration Medical Center, Miami, FL 33125, USA

## Abstract

*Purpose*. To describe a case of 3 refractive procedures performed in one eye over 2 decades. *Methods*. Case report. *Results*. A 41-year-old patient presented for refractive surgery evaluation. His ocular history includes bilateral radial keratotomy performed 21 years ago for moderate myopia: spherical equivalence of −4.25 D bilaterally. Postoperative uncorrected visual acuity (UCVA) was 20/30; however, over time he developed a hyperopic shift and UCVA decreased to 20/40 in the right eye. Thus, laser-assisted in situ keratomileusis (LASIK) was performed at an outside institution 6.5 years later, and the patient had initial improvement of UCVA to 20/25. Due to a change in refractive error, the patient underwent uneventful astigmatic keratotomy 13 years after LASIK in the right eye, and 1.5 years after surgery best-corrected visual acuity was 20/25 with manifest refraction of −1.00 + 5.50 × 134°.  *Conclusion*. We report the outcomes and natural refractive progression in a patient who underwent three corneal refractive procedures over two decades. This case underlines the difficulties of long-term management of post-RK hyperopia and astigmatism.

## 1. Introduction

Radial keratotomy (RK), first performed in the United States in 1978, was viewed as an effective procedure with excellent immediate postoperative outcomes [1]. However, it has been well documented that the radial incisions made in the cornea cause gradual hyperopic shifts. The causes of such hyperopic shift after RK are not well known, but these shifts have been speculated to be due to peripheral corneal bulging and compensatory central corneal flattening [2]. Such incisional alterations cause biomechanical instability of the cornea, leading to unpredictable long-term results [3–5]. Laser-assisted in situ keratomileusis (LASIK) has been used in these cases, but results are suboptimal in patients with hyperopic astigmatism and long-term results are not as stable or as predictable as in naïve corneas [6–8]. Astigmatic keratotomy (AK) has been described in patients with naïve corneas and with postpenetrating keratoplasty to correct astigmatism, but it has not been discussed after RK to our knowledge. We describe outcomes after LASIK and AK were performed sequentially in the same eye to correct post-RK hyperopia and astigmatism to highlight the difficulties in managing refractive status in such patients.

## 2. Case Presentation

**Figure 1:**
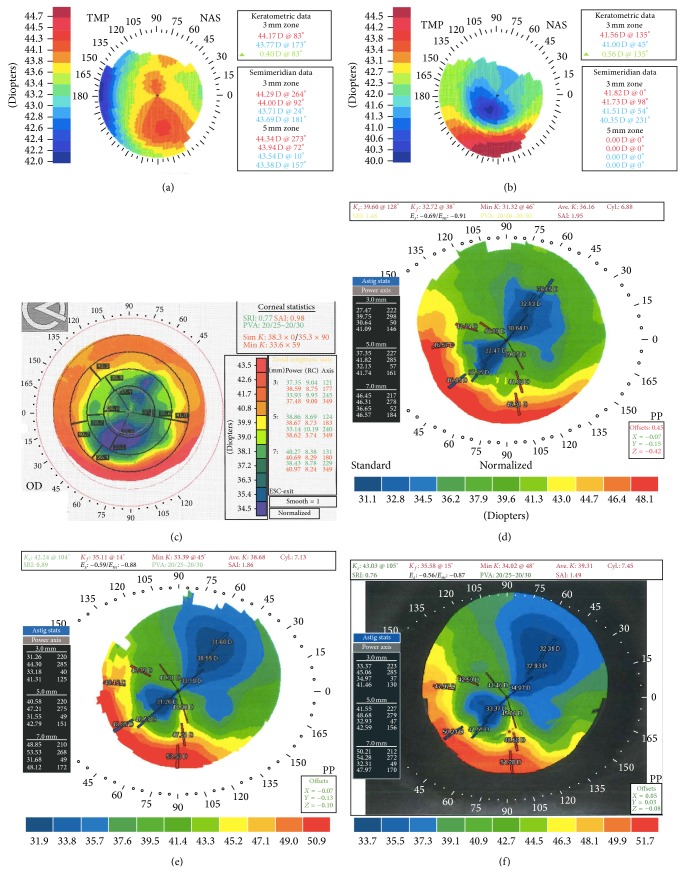
Corneal Topography. Progression of corneal topography measured in patient's right eye: (a) preoperative topography (before radial keratotomy [RK]), (b) postoperative month 1 after RK, (c) postoperative month 5 after LASIK, (d) postoperative year 6 after LASIK, (e) postoperative month 4 after astigmatic keratotomy (AK), and (f) postoperative year 1.5 after AK.

A 41-year-old Hispanic man who wore soft contact lenses for 15 years presented to Bascom Palmer Eye Institute for refractive surgery evaluation 21 years ago. In the right eye, uncorrected visual acuity (UCVA) was 6/200 and manifest refraction (MR) was −4.5 sphere with corrected distance visual acuity (BCVA) 20/20. Preoperative topography showed a low amount of with-the-rule astigmatism (corneal cylinder (CC) = 0.40 D; Figure 1(a)). In the left eye, initial UCVA was 7/200 and MR was −4.5 sphere with BCVA 20/20. Preoperative topography showed a low amount of with-the-rule astigmatism (CC = 0.73 D). To correct this patient's refractive error, RK was performed bilaterally.

**Figure 2:**
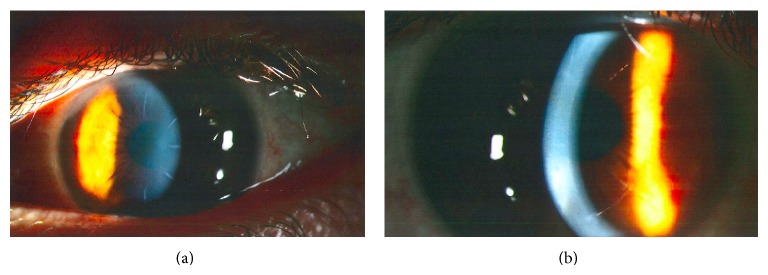
Slit Lamp Photography. Patient's right eye detailing: (a) radial keratotomy incisions and (b) astigmatic keratotomy incision. Even though the patient had LASIK, it is difficult to appreciate the LASIK flap in these photos.

For RK, ultrasound pachymetry measured a corneal thickness of 554 *μ*m at a position 1.5 mm temporal to the center of the cornea. The diamond blade was set to 544 *μ*m. With the diamond knife, 8 centripetal manual incisions were created in a Russian-order fashion [9], leaving a 4 mm optic zone (Figure 2(a)). On postoperative day (POD) 1, the patient had UCVA of 20/30 and BCVA of 20/25 in the right eye. On postoperative month (POM) 1, UCVA was stable (Figure 1(b)). Two years postoperatively, UCVA was 20/20, but the patient complained of increasing ghost images, haloes, and double vision. Over 6 years, the patient developed a hyperopic shift in the right eye (UCVA 20/40; MR +1.25 sphere with BCVA 20/25; CC = 1.50 D). However, in the left eye, visual acuity remained stable and clear, without hyperopic shift or astigmatism (UCVA 20/20; MR −0.25 + 0.25 × 65° with BCVA 20/20; CC = 0.87 D).

To address the hyperopic shift in the right eye, LASIK was performed at an outside institution 6.5 years after the RK procedure. A 130 *μ*m flap was created with microkeratome and excimer ablation was performed with VisX Star (Abbott Medical Optics, Santa Ana, CA, USA). On POD-4, UCVA was 20/25 and MR was +0.75 sphere with BCVA 20/20. Five months after LASIK, UCVA was 20/30 and MR was +0.75 sphere with BCVA 20/20, but the patient complained of haloes and double vision. Three and a half years after LASIK and according to medical records, the patient presented with worsening VA (UCVA 20/50; MR of +1.25 + 2.75 × 119° with BCVA 20/20), which continued to deteriorate over the next 3 years (UCVA 20/400; MR −1.50 + 5.50 × 152° with BCVA 20/20; topography Figures 1(c) and 1(d)). Seven years later, the change in BCVA and refractive error was mild (UCVA 20/300; MR −2.25 + 6.50 × 135° with BCVA 20/25).

Thirteen years after his LASIK procedure, the patient returned to our institution complaining of decreased visual acuity. He tried contact lenses but proved intolerant with poor contact lens hygiene and insisted on being less dependent upon glasses; therefore an additional refractive procedure was considered to reduce astigmatism as a last option before a more invasive procedure (such as corneal transplant) would be considered. In order to correct this residual astigmatism, femto-AK was performed using the IntraLase 150 kHz femtosecond laser (Abbott Medical Optics, Santa Ana, CA, USA). Two arcuate incisions were made (135° and 315°), each 70° in length, at a depth of 412 *μ*m (Figure 2(b)). On POD-1, UCVA was 20/25 and MR was plano +0.50 × 137° with BCVA 20/25. On POM-4, BCVA was 20/25 and increased cylinder was noted (MR −1.00 + 6.00 × 128°, CC = 7.13 D, Figure 1(e)). As such, the incisions were slightly opened at the slit lamp using a 30-gauge needle. A year and a half later, UCVA was 20/50 and astigmatism was stable (MR −1.00 + 5.50 × 134° with BCVA 20/20; Figure 1(f)).

On the other hand, in the left eye, although the patient developed a hyperopic shift with mild increasing astigmatism, he did not undergo any further refractive procedures and was comfortable wearing rigid gas permeable contact lenses in this eye (UCVA 20/50, MR +4.25 + 1.75 × 50° with BCVA 20/40; CC = 1.76).

## 3. Discussion

This case demonstrates the complexity of managing post-RK refractive outcomes. Visual disturbances, hyperopia, and astigmatism are well documented in post-RK patients. Visual dissatisfaction with RK has many etiologies, including corneal scarring, irregular astigmatism, hyperopic shift, and increasing width of radial incisions over time with resultant visual fluctuations [3, 4, 7]. As commonly seen, our patient developed hyperopic shift and astigmatism after RK in both eyes. To improve his visual acuity, LASIK and AK were performed in the right eye, 6.5 and 13 years later, respectively.

Results of LASIK to correct post-RK residual refractive error have been mixed: some studies showed relatively good outcomes, whereas others demonstrated poor outcomes and increased complication rates. For example, a study by Lipshitz et al. (12 eyes, 80%) and Francesconi et al. (55 eyes, 80%) demonstrated that post-RK eyes had refractive error within ±1.00 D of emmetropia 7 months after LASIK [6, 8]. However, recurrent epithelial ingrowth leading to irregular astigmatism and loss of best-corrected vision have been reported [10]. Moreover, LASIK after RK proves to be more challenging than LASIK after other refractive procedures due to the fact that LASIK incision after RK must cut through the RK incisions. Therefore, the risk of flap and incision related complications are higher in these patients [11]. PRK is a surface ablation technique that avoids the creation of flaps and thus all flap-related complications. Hence, PRK is our current preferred technique in post-RK eyes, even though the associated risks include postoperative regression, haze, and unpredictable visual outcomes [12, 13]. Furthermore, such procedures (LASIK and PRK) are less predictable with poorer outcomes in corneas with irregular astigmatism, as the algorithms that are used to design treatment are meant for corneas with regular astigmatism. A better option for our patient might have been topography-guided surface treatment due to the irregular pattern of his astigmatism; however such techniques are not FDA-approved in the United States.

While AK is a good procedure to treat higher levels of astigmatism, to our knowledge, no cases have previously discussed this technique in the setting of post-RK-post-LASIK astigmatism. In our patient, astigmatism initially improved but these preliminary results were not stable and the patient had regression to his original degree of astigmatism. An option for post-RK visual changes or corneal ectasia includes corneal collagen cross-linking, which restores corneal stability. A case report by Mazzotta et al. reported CXL performed 10 years after RK due to corneal ectasia and hyperopic shift and at 1-year follow-up UCVA improved from 20/100 to 20/30 Snellen lines [14]. Another study by Elbaz et al. reported 9 eyes that underwent post-RK CXL due to worsening visual acuity; however the resulting 1 year follow-up UCVA was not statistically significantly different (20/160 to 20/80, *P* = 0.3) [15]. However, long-term prospective studies are needed to validate this method.

To conclude, this case reinforces the difficulties in the long-term management of post-RK hyperopia and astigmatism. While not perfect, LASIK and AK provided visual rehabilitation in this patient, who had poor contact lens hygiene and wished to be less dependent upon glasses. Although topographically the outcome was not ideal, the patient was happy with his uncorrected visual outcome and was able to function in his daily activities without contact lenses after the AK procedure. However, the limitations of each procedure need to be stressed during the preoperative evaluation and realistic visual expectations need to be emphasized in these difficult cases.

## References

[1] Wang M. (2000). History of refractive surgery. *LASIK Vision Correction: The Exciting New Laser Eye Surgery*.

[2] Hanna K. D., Jouve F. E., Waring G. O. (1989). Preliminary computer simulation of the effects of radial keratotomy. *Archives of Ophthalmology*.

[3] Waring G. O., Lynn M. J., McDonnell P. J. (1994). Results of the prospective evaluation of radial keratotomy (PERK) study 10 years after surgery. *Archives of Ophthalmology*.

[4] Waring G. O., Lynn M. J., Culbertson W. (1987). Three-year results of the prospective evaluation of radial keratotomy (PERK) study. *Ophthalmology*.

[5] Deitz M. R., Sanders D. R., Raanan M. G., DeLuca M. (1994). Long-term (5- to 12-year) follow-up of metal-blade radial keratotomy procedures. *Archives of Ophthalmology*.

[6] Lipshitz I., Man O., Shemesh G., Lazar M., Loewenstein A. (2001). Laser in situ keratomileusis to correct hyperopic shift after radial keratotomy. *Journal of Cataract & Refractive Surgery*.

[7] Rowsey J. J., Balyeat H. D. (1982). Preliminary results and complications of radial keratotomy. *The American Journal of Ophthalmology*.

[8] Francesconi C. M., Nosé R. A. M., Nosé W. (2002). Hyperopic laser-assisted in situ keratomileusis for radial keratotomy-induced hyperopia. *Ophthalmology*.

[9] Casebeer J. C., Rae C. A. (1993). Keratorefractive diamond blade and surgical method. *Google Patents*.

[10] Forseto A. S., Nosé R. A. M., Francesconi C. M., Nosé W. (1999). Laser *in situ* keratomileusis for undercorrection after radial keratotomy. *Journal of Refractive Surgery*.

[11] Peacock L. W., Slade S. G., Martiz J., Chuang A., Yee R. W. (1997). Ocular integrity after refractive procedures. *Ophthalmology*.

[12] Joyal H., Grégoire J., Faucher A. (2003). Photorefractive keratectomy to correct hyperopic shift after radial keratotomy. *Journal of Cataract and Refractive Surgery*.

[13] Kuo I. C., Lee S. M., Hwang D. G. (2004). Late-onset corneal haze and myopic regression after photorefractive keratectomy (PRK). *Cornea*.

[14] Mazzotta C., Baiocchi S., Denaro R., Tosi G. M., Caporossi T. (2011). Corneal collagen cross-linking to stop corneal ectasia exacerbated by radial keratotomy. *Cornea*.

[15] Elbaz U., Yeung S. N., Ziai S. (2014). Collagen crosslinking after radial keratotomy. *Cornea*.

